# Integrated physiological, metabolomic, and proteome analysis of *Alpinia officinarum* Hance essential oil inhibits the growth of *Fusarium oxysporum* of *Panax notoginseng*

**DOI:** 10.3389/fmicb.2022.1031474

**Published:** 2022-11-16

**Authors:** Xiao-Yun Liu, Ying-Ying Huo, Jing Yang, Tian-Tian Li, Fu-Rong Xu, He-Ping Wan, Jia-Nan Li, Chun-Hong Wu, Yong-Hong Zhang, Xian Dong

**Affiliations:** ^1^School of Chinese Materia Medica, Yunnan University of Chinese Medicine, Kunming, China; ^2^College of Life Sciences, Hubei Engineering Research Center for Protection and Utilization of Special Biological Resources in the Hanjiang River Basin, Jianghan University, Wuhan, China; ^3^Laboratory of Medicinal Plant, Institute of Basic Medical Sciences, School of Basic Medicine, Biomedical Research Institute, Hubei Key Laboratory of Wudang Local Chinese Medicine Research, Hubei University of Medicine, Shiyan, China

**Keywords:** essential oil, steroid biosynthesis, tricarboxylic acid cycle, cell cycle and meiosis, antifungal mechanism

## Abstract

*Fusarium oxysporum* is the main pathogen of *Panax notoginseng* root rot, and chemical fungicides remain the primary measures to control the disease. Plant essential oil (EO) is a volatile plant secondary metabolic product that does not produce any residue to replace chemical pesticide. To comprehensively understand the antifungal mechanism of *Alpinia officinarum* Hance EO, the physiological indicators, proteome and metabolome were analyzed using *F. oxysporum* spores and hyphae treated with different EO concentrations. The cell membrane was damaged after both low and high concentrations of EO treatment, along with leakage of the cell contents. To resist the destruction of membrane structure, fungi can increase the function of steroid biosynthesis and expression of these catalytic enzymes, including squalene monooxygenase (SQLE), sterol 14alpha-demethylase (CYP51, CYP61A), delta14-sterol reductase (TM7SF2, ERG4), methylsterol monooxygenase (MESO1), and sterol 24-C-methyltransferase (SMT1). Furthermore, the tricarboxylic acid cycle (TCA) was influenced by inhibiting the expression of glutamate synthase (GLT1), 4-aminobutyrate aminotransferase (ABAT), and succinate-semialdehyde dehydrogenase (gabD); increasing malate and gamma-aminobutyric acid (GABA); and decreasing citrate content. The spore germination rate and mycelia growth were decreased because the expression of cohesin complex subunit SA-1/2 (IRR1) and cohesion complex subunit (YCS4, BRN1, YCG1) were inhibited. Particularly, under high EO concentrations, cyclin-dependent kinase (CDC28) and DNA replication licensing factor (MCM) were further inhibited to disrupt the cell cycle and meiosis, thus affecting cell division. The results of this study will enrich the understanding of the antifungal mechanism of EOs and provide an important basis to develop new plant-derived fungicides.

## Introduction

*Panax notoginseng* (Burk.) F. H. Chen is a perennial herb of the *Panax* genus, whose roots improve hemostasis and promote blood circulation (Wang et al., 2016). Approximately 100 Chinese patent medicines are available with *P. notoginseng* as the raw material. To meet the consumer’s growing demand, all of the *P. notoginseng* sold on the market is artificially cultivated for 3–5 years. *P. notoginseng* is suitable for growth in semi shade and humid ecological environments and is a subtropical mountain shade-loving plant with a narrow ecological range (Hong et al., 2010). Because of its special habitat requirements, continuous cropping disorders are becoming frequent, and *Fusarium oxysporum* is a primary pathogen of *P. notoginseng* root rot. Fusarium infection can damage crop yield and cause mycotoxin contamination, some of which seriously threaten animal and human health. Presently, the occurrence of *P. notoginseng* root rot seriously threatens the development of the health industry.

The prevention and control methods for this disease include agricultural, physical, chemical and biological control, among which chemical control is the most commonly used method, such as hymexazol, carbendazim, and chlorothalonil. Chemical control plays a crucial role in disease control but has many side effects. The long-term application of chemical fungicides makes pathogens produce certain resistance, making it more difficult to control diseases (Villa et al., 2017). While preventing and controlling diseases, the survival of many other organisms will also be threatened to a certain extent, and biodiversity will be severely affected. Additionally, some chemical pesticides that are difficult to degrade can accumulate step by step through the food ecological chain and enter the human body, subsequently triggering different metabolic pathways of the human body and potentially harming human health. In recent years, with increasing environmental protection awareness, consumers have increasingly higher requirements for the nutrition and safety of agricultural products. The era of simply pursuing the quantity of agricultural products has now passed. Ensuring quantity, high-quality and pollution-free development has become the goal of people’s daily lives. Therefore, plant-derived pesticides have gradually become the focus of modern pesticide research (Isman et al., 2010).

Studies have shown that aromatic plant essential oil (EO) has bactericidal and insecticidal abilities (Pandey et al., 2016) and can be metabolized by the environment with little impact on biology and the environment. Therefore, it is a new generation of plant-derived pesticides with great potential for development (Perricone et al., 2020). Plant EO is a class of aromatic secondary metabolites that widely exist in aromatic plants and contains different substances, such as alcohols, aldehydes, esters, and phenols (Koul, 2008). Plant EO is a mixture of plant secondary metabolites, and the complexity of its components is closely related to plant species, growth conditions, collection sites and harvest seasons. The complex diversity of the EO composition makes it possess broad antimicrobial activity because of its inhibitory effect on multiple targets of pathogens. Currently, some achievements have been made in the study of antimicrobial mechanisms. Studies have reported that plant EO and its main components progressively destroy fungal pathogens: mycelial morphology changes, vacuolar fusion, cell wall fiber layer shedding and organelle destruction. With the widespread degradation and dissolution of nuclei and mitochondria, the cytoplasmic contents disintegrate, leading to the death of the pathogen (Shokri, 2016; Nascimento et al., 2019).

Because of the lack of systematic research on the antimicrobial mechanism of EOs, developing new green pesticides has substantial blindness and randomness. This study aimed to (1) evaluate the inhibitory effect of *A. officinale* EO using the physiological index of *F. oxysporum* and (2) decipher the mechanisms of the antimicrobial effects of *A. officinale* EO on *F. oxysporum* by integrated proteomics and metabolomics analysis. Based on these data, we expect to further develop and use EOs to produce environmentally friendly pesticides or fertilizers to alleviate root rot disease in medicinal plants.

## Materials and methods

### Plant material and EO extraction

*A. officinale* was purchased from Yunnan Jinfa Co., Ltd. (Kunming, China) and was identified by Professor Cheng Yong-xian. EO was obtained by steam distillation (Ma et al., 2019). The extraction process involved adding the herb and pure water at a ratio of 1:8 (v/v) and extracting for 6 h. The obtained distillate was dried with anhydrous sodium sulfate to remove excess water and stored in brown glass bottles at −20°C until analysis. The chemical constituents of *A. officinarum* were analyzed using GC–MS (Agilent Technologies; 7890B-5977B) according to our previously described method (Chen et al., 2020).

### Root rot pathogen

The pathogen was isolated from the root rot of *P. notoginseng* and identified as *F. oxysporum* by amplifying the ITS fragment with ITS1/ITS4 universal primers. The pathogen was purified by single spore purification and preserved in potato dextrose agar (PDA) medium.

### Determination of spore germination

After *F. oxysporum was* cultured on PDA medium for 7 days, mycelium fragments were placed into 500 ml Erlenmeyer flasks containing Bilay’s culture medium (Tan et al., 2011). The culture was rotated at 180 rpm/min in a shaker at 28°C for 5 days. After removing the mycelia through four layers of cheesecloth, the spores were collected and washed twice with sterile water. The spore concentration was quantified using a hemocytometer and then was adjusted to a 10^6^ spore suspension/ml. The minimum inhibitory concentration (MIC) of EO was determined by the method published by our research group (Ma et al., 2019). The MIC (M), half of the MIC (M0.5), a quarter of MIC (M0.25) and an eighth of the MIC (M0.125) were, respectively, prepared. The negative control was 2-DMSO-T (20/1000 DMSO and 1/1000 Tween-80 suspension), which dissolved the EO. One milliliter of spore suspension was mixed with 1 ml of the above prepared *A. officinarum* EO and 2-DMSO-T as the treatment and negative control groups, respectively. The mixture was placed in an incubator at 28°C in the dark for 24 h. Each treatment had 3 replicates. The spore suspension was placed on a glass slide and observed under a microscope. More than 5 fields were randomly selected, and the number of germinated spores was counted. When the spore germination tube length was greater than 1/2, it was regarded as spore germination (Plascencia-Jatomea et al., 2010).

Spore germination rate (%) = (spore germination/total spore microscopic number) × 100%.

### Preparation of mycelia and *Alpinia officinarum* EO mixture

The spores of *F. oxysporum* were inoculated into YEPD medium and cultured at 180 rpm/min and 28°C for 4 days. The mycelia were filtered through four layers of cheesecloth and washed 2–3 times in sterile PBS solution to obtain mycelia. A quantitative amount of mycelium (2 g) was placed into a 50 ml triangulated bottle containing a 40 ml mixture of *A. officinarum* EO and sterile purified water. The final concentration of *A. officinale* EO reached M, M0.5, M0.25 and M0.125. The control group was treated with the same amount of 2-DMSO-T and hymexazol (chemical pesticide). The triangulated bottle was sealed with sealing film to prevent EO volatilization. Each treatment had three biological duplications and was repeated three times.

### Determination of the fresh weight and dry weight of *Fusarium oxysporum* mycelia

Five milliliters of suspension prepared as 2.4 was taken at 0, 6, 12, 24, 48, 72, 96, and 120 h into a 10 ml centrifuge tube with accurate weighing. After centrifugation at 8500 rpm/min for 5 min, the supernatant was discarded and the centrifuge tube containing mycelium precipitation was accurately weighed. The difference before and after placing the suspension in the centrifuge tube was the fresh weight of mycelium. The centrifuge tube after weighing was placed in the oven to dry at 45°C to constant weight, and the dry weight was recorded.

### Determination of membrane permeability

The mycelia and *A. officinarum* EO mixture to be tested was prepared as described above (Section 2.4). The membrane permeability of the mycelia was determined by measuring the relative electrical conductivity (Yan et al., 2010). Briefly, the conductivity of each treatment was measured in the absence of mycelium (denoted as V_0_); after adding mycelium, the conductivity was measured at 0, 0.5, 1, 1.5, 2, 2.5, 3, 3.5, 4, 4.5, 5, 5.5, 6, 6.5, 7, and 7.5 h (denoted as V_1_). Finally, the liquid from each treatment was boiled in hot water for 5 min, and the conductivity was measured after cooling to room temperature (denoted as V_2_).

Relative conductivity = (V1−V0)/V_2_ × 100%.

### Extracellular protein content and soluble reducing sugar content of *Fusarium oxysporum* mycelia

Mycelia collection and treatment were the same as above 2.4. Five milliliters of the mixture was taken at 0, 6, 12, 24, 48, 72, 96, and 120 h and then centrifuged at 4°C at 8500 rpm/min for 5 min. The soluble protein content of the supernatant was determined using Coomassie brilliant blue G-250 staining (Hatada et al., 2006). Specifically, 200 μl of supernatant was mixed with 1 ml of Coomassie brilliant blue G-250 staining reagent, and the absorbance was measured at 595 nm after 2 min of reaction. According to the standard curve of the protein content, the extracellular protein content of *F. oxysporum* mycelia was obtained. Each treatment had 4 replicates.

The content of extracellular reducing sugars was determined using the colorimetric method of 3,5-dinitrosalicylic acid (Hatada et al., 2006). Five hundred microliters of the above supernatant was mixed with 0.5 ml of 3,5-dinitrosalicylic acid solution, followed by incubation in boiling water for 5 min. After cooling to room temperature, 4 ml of distilled water was added, and the absorbance of the mixture was measured at 595 nm. The extracellular reducing sugar content of *F. oxysporum* mycelia was calculated based on the standard curve of reducing sugar.

### Scanning and transmission electron microscopy

When *F. oxysporum* mycelia were treated with different concentrations (0, M0.5 and M) of *A. officinarum* EO for 12 and 24 h, the mycelia were filtered using 4 layers of gauze. After adding 2.5% glutaraldehyde buffer, the cells were fixed at 4°C for more than 12 h, rinsed with 0.1 mol/l PDB buffer and dehydrated with gradient alcohol. The surface and internal structure of mycelium samples were scanned (S-3000 N; Hitachi, Co. Ltd., Tokyo, Japan) and transmitted (JEM-1011; JEOL, Co. Ltd., Tokyo, Japan) to observe the changes in the microscopic morphology and structure.

### Proteomics and metabolomics analysis

To reveal the antifungal mechanism of *A. officinarum* EO, proteomics and metabolome analyses of *F. oxysporum* were conducted. The hyphae of *F. oxysporum* were treated with different concentrations of *A. officinarum* EO (0, M0.5 and M) for 24 h. The mycelia were cleaned with PBS buffer solution, and the liquid on the mycelia surface was adsorbed onto disinfected filter paper. The collected mycelia were placed into a cryotube, flash-frozen with liquid nitrogen for 5 min, and immediately stored in a-80°C ultralow temperature refrigerator for subsequent proteome sequencing. Three biological duplications were performed for each group.

For metabolomics analysis, 1 g of the aboveground mycelia sample was weighed and frozen in liquid nitrogen. Sample preparation for the metabolome was performed at Wuhan Matwell Biotechnology Co., Ltd. (Wuhan, China) using standard procedures, and three biological replicates of each treatment were analyzed. Metabolites of *F. oxysporum* were analyzed using a UPLC–ESI–MS/MS system (UPLC: SHIMADZU Nexera X2^1^; MS: Applied Biosystems 6500 Q TRAP^2^). ESI Turbo Ion-Spray interface of mass spectrometry equipment, which is controlled by Analyst 1.6.3 software in the positive and negative ion running mode, was used and the data were collected in the negative ion mode. The metabolites were analyzed by mass spectrometry based on the MWDB (Metware Database) of Wuhan Matwell Biotechnology Co., Ltd. Metabolite data were analyzed using multivariate statistical analysis to improve normality and normalization (Carlos et al., 2010). Principal component analysis (PCA) and hierarchical cluster analysis (HCA) were performed using R software^3^. The screening criteria for differentially accumulated metabolites (DAMs) were *p* < 0.05, fold change≥2 and VIP (variable importance in projection) ≥ 1. Finally, the KEGG database was used for pathway enrichment analysis of DAMs.

Sample preparation for the proteomics analysis was performed at Hangzhou Jingjie Biotechnology Co., Ltd. (Hangzhou, China) using standard procedures (Yu et al., 2022), and three biological replicates of each treatment were analyzed. After a first-order mass spectrometry acquisition, the second-order spectrogram with the charge number of parent ions in the range of 0–5 was collected in 10 scans in the PASEF mode. Secondary mass spectrometry data were retrieved using MaxQuant 1.6.15.0. Regarding the retrieval parameter settings, the database was Fusarium oxysporum_5507 (246,924 sequences), and an inverse database was added to calculate the false-positive rate (FDR) caused by random matching. Additionally, a common contamination database was added to eliminate the influence of contaminated proteins in the identification results. The number of missed cuts was set to 2. The minimum peptide length was set to 7 amino acid residues. The maximum modification number of the peptide was set to 5. The mass error tolerance of both the primary parent ion and secondary fragment ion was set at 20 PPM. When the *p* value was <0.05 and the differential expression level changed by more than 1.5 times, proteins containing at least 2 peptides were selected as differential proteins.

Gene Ontology (GO) enrichment analysis and Kyoto Encyclopedia of Genes and Genomes (KEGG) pathway analysis were performed for the differentially expressed proteins. GO was derived from the UniProt-Goa database^4^. The KEGG database was used to annotate the protein pathways.

### RNA extraction and quantitative real-time (qRT)-PCR

Total RNA was isolated using Trizol isolation reagent, and first-strand cDNA was synthesized from 1 g of total RNA using the Transcriptor Fist Strand cDNA Synthesis Kit (Transgene Biotech) by following the protocol. qRT-RCR were run in three biological replicates and 3 technical replicates. For the normalization of gene expression, the actin gene (FOXG_12791) was used as an internal standard, and the non-treated hypha was used as control.

### Statistical analysis

The data are presented as the mean values ± standard deviations across five repeated measurements. Statistical significance was using OneWay ANOVA and Duncan’s multiple comparisons test at *p* ≤ 0.05 versus the control.

## Results

### Effect of *Alpinia officinarum* EO on spore germination of *Fusarium oxysporum*

**Figure 1:**
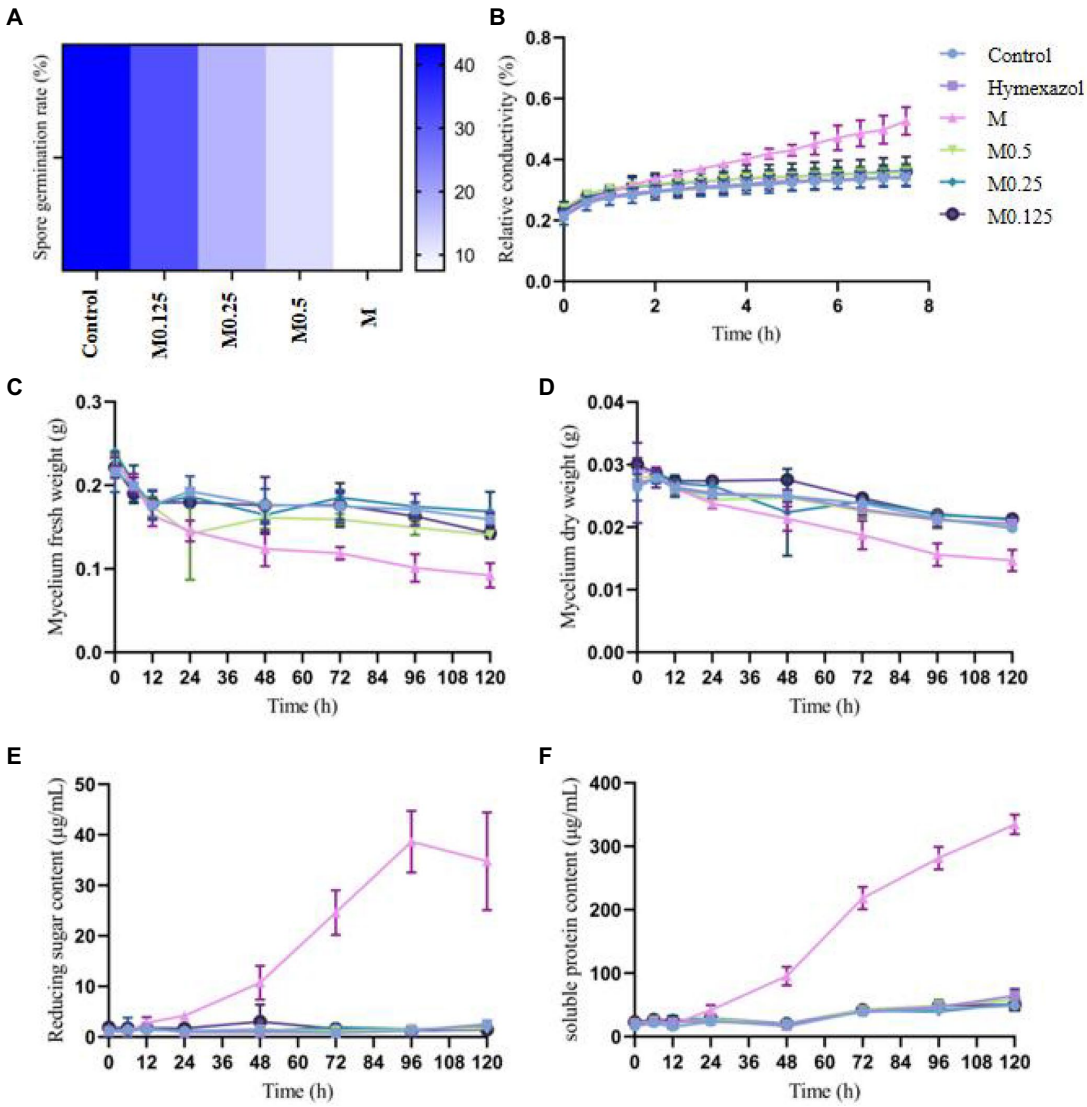
Effects of different concentrations *of Alpinia officinarum* essential oil on the spore germination **(A)**, cell membrane permeability **(B)**, fresh weight **(C)**, dry weight **(D)**, reducing sugar content **(E)** and soluble protein content of *F. oxysporum* **(F)**. 2-DMSO-T (20/1000 DMSO and 1/1000 Tween-80 suspension) was used as a negative control (CK), and Hymexazol was used as a positive control. The minimum inhibitory concentration (MIC, abbreviation for M) of EO was determined previously to be 1.12 mg/ml. Various *A. officinale* EO concentration gradients (M, M0.5, M0.25, M0.125) were prepared. M0.5: half concentration of M; M0.25: one-quarter of M; M0.125: one-eighth of M.

The MIC of *A. officinarum* EO against *F. oxysporum* was 1.12 mg/ml. The spore germination rate of *F. oxysporum* decreased with increasing EO concentration. After 24 h of culture, the spore germination rate of the control group was 43.12%, while those of the M0.125 group, M0.25 group, M0.5 group and M group were 32.21, 17.76, 12.12 and 7.49%, respectively. Compared with the control group, except for the M0.125 group, the spore germination rate in the other groups decreased significantly (Figure 1A). The spore germination rate in the M group was 5.76 times lower than that in the control group. However, when the concentration was reduced to 0.14 mg/ml (M0.125), the spore germination rate was slightly lower than that in the control group, but the difference was not statistically significant.

### Effects of *Alpinia officinarum* EO on the relative electrical conductivity of *Fusarium oxysporum* mycelia

The cell membrane has various physiological functions. When the cell membrane is damaged, its normal physiological metabolism cannot be performed, and leakage of the cell contents leads to increased conductivity of extracellular fluid. After treatment with the MIC of *A. officinarum* EO, the extracellular relative conductivity of its mycelium increased significantly at 2.5 h compared with the control group and gradually increased with the extension of treatment time. No significant increase was found in the extracellular relative conductivity in the other groups (Figure 1B).

### Effect of *Alpinia officinarum* EO on the fresh and dry weight of *Fusarium oxysporum* mycelia

After treating *F. oxysporum* mycelia with different concentrations of *A. officinarum* EO, the fresh weight of mycelia in the M group decreased significantly at 48 h; no significant difference was found among the other groups (Figure 1C). The dry weight of mycelia in the M group decreased significantly at 72 h compared with that in the other groups; no significant difference was found among the other groups (Figure 1D). Additionally, with increasing treatment time, the mycelial fresh weight and dry weight of the M group gradually decreased.

### Effects of *Alpinia officinarum* EO on the leakage of *Fusarium oxysporum*

Damage to the cell membrane will lead to release of the cell contents, such as proteases, nucleic acids and other macromolecules, as well as ions and sugars. Additionally, reducing substances released by mycelium during lysis can be detected as reducing sugars. The extracellular soluble protein and reducing sugar indicated the damage degree of *A. officinarum* EO to the cell membrane and leakage degree of the cell contents. Compared with the control group, the extracellular reducing sugar and soluble protein contents of *F. oxysporum* treated with the MIC of *A. officinarum* EO significantly increased after 24 h and 48 h, while no significant increase was found in the other groups (Figures 1E,F). The contents of the mycelium of *F. oxysporum* were continuously released after treatment with *A. officinarum* EO for 24 h, and the membrane of the mycelium might have been damaged.

### Effects of *Alpinia officinarum* EO on the mycelial morphology and cellular structure of *Fusarium oxysporum*

**Figure 2:**
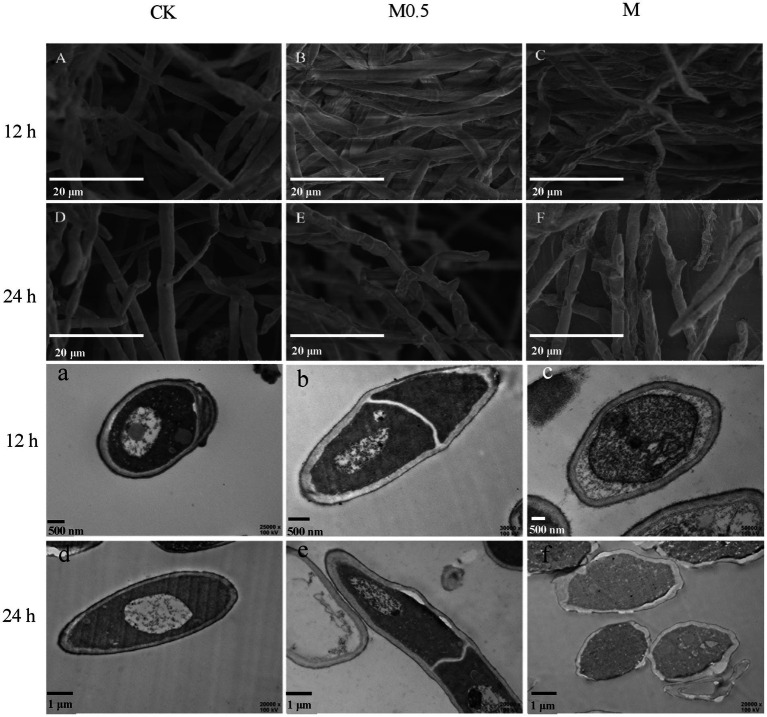
Scanning electron micrographs **(A–F)** and transmission electron microscopy **(a–f)** of *F. oxysporum* treated with different concentrations of *A. officinarum* Hance EO for 12 h and 24 h, respectively. (**A–C**, **a–c**): EO treatment for 12 h; (**D–F**, **d–f**): EO treatment for 24 h. All treatments were consistent with the above.

The morphological structure of *F. oxysporum* mycelia treated with *A. officinarum* EO was observed by scanning electron microscopy and transmission electron microscopy (Figure 2) to further explore the antifungal mechanism of *A. officinarum* EO against *F. oxysporum*. In the control group, a smooth surface and full mycelia were observed (Figure 2A,D). However, after *A. officinarum* EO was added, the mycelia were dry, wrinkled and partially damaged. When treated with *A. officinarum* EO for 12 h, mycelium in the M0.5 group showed slight shrinkage (Figure 2B), while mycelium in the M group showed severe shrinkage (Figure 2C). When mycelium was treated for 24 h, mycelium in the M0.5 group was severely shrunken (Figure 2E), and the mycelium surface in the M group was severely damaged (Figure 2F). With increasing EO concentration and treatment time, the degree of shrinkage and damage of *F. oxysporum* mycelium gradually increased (Figure 2). The scanning electron microscopy results showed that *A. officinarum* EO substantially changed the mycelial morphology of *F. oxysporum*.

With increasing treatment time and concentration of EO, the damage degree of the cell wall and cell membrane of *F. oxysporum* gradually increased, and the organelle boundary was gradually blurred (Figure 2). In the M0.5 group, plasma wall separation occurred at 24 h (Figure 2e); in the M group, the apparent morphology and internal subcellular structure of mycelium were significantly damaged. Additionally, the organelles and boundaries were unclear, and various contents in the cell lumen were mixed (Figure 2f). Scanning electron microscopy (SEM) and transmission electron microscopy (TEM) images showed that *A. officinarum* EO could damage the cell membrane and cell wall of *F. oxysporum* mycelium, and the extent was dependent on the concentration and treatment time of EO.

### Characterization of the metabolome of *Fusarium oxysporum* of *Panax notoginseng*

**Figure 3:**
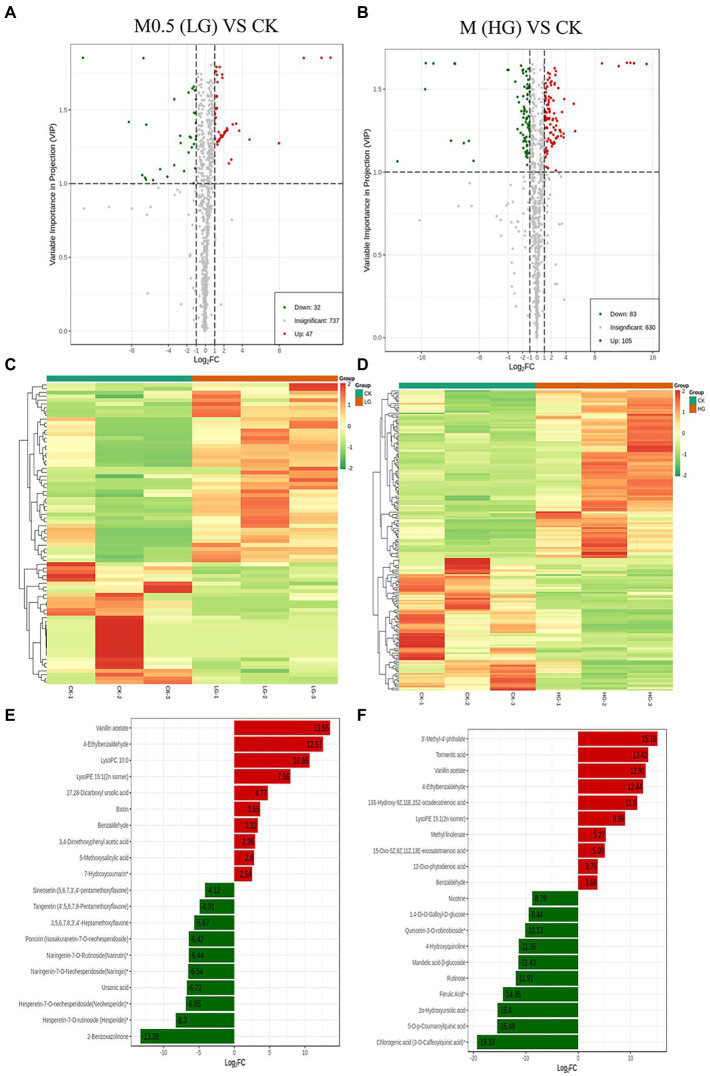
Up- and downregulation of differentially accumulated metabolites (DAMs) in two comparison groups. Volcano plot **(A)** and heatmap **(C)** of DAMs in M0.5 (LG) vs. CK. Volcano plot **(B)** and heatmap **(D)** of DAMs in M (HG) vs. CK. The top ten DAMs in M0.5 (LG) vs. CK **(E)** and in M (HG) vs. CK **(F)**, respectively. Each dot in the volcano plot denotes one metabolite. The green spots denote metabolites with down-regulated differential expression; the red spots denote metabolites with up-regulated differential expression; the gray spots denote the detected metabolites without significant differences. All treatments were consistent with the above.

A total of 819 metabolites were analyzed from the two groups of samples, with 79 differentially accumulated metabolites (DAMs) obtained in M0.5 (LG) vs. CK, 32 upregulated and 47 downregulated DAMs; M vs. CK comprised 83 upregulated and 105 downregulated DAMs (Figures 3A,B). A heatmap of DAMs was constructed, and the DAMs in these two comparison groups reflected the abovementioned changes (Figures 3C,D). According to the fold changes in the accumulation of the metabolites, we identified the top ten DAMs that increased or decreased in each comparison group (Figures 3E,F).

The metabolite contents were normalized to construct a hierarchical clustering heatmap (Supplementary Figure S3A). Principal component analysis (PCA) was subsequently performed, and the results showed significant separation between the HG samples and the other two samples. PC1 and PC2 explained more than 52% of the variability and were mainly distinguished by PC1 (Supplementary Figure S4B).

**Figure 4:**
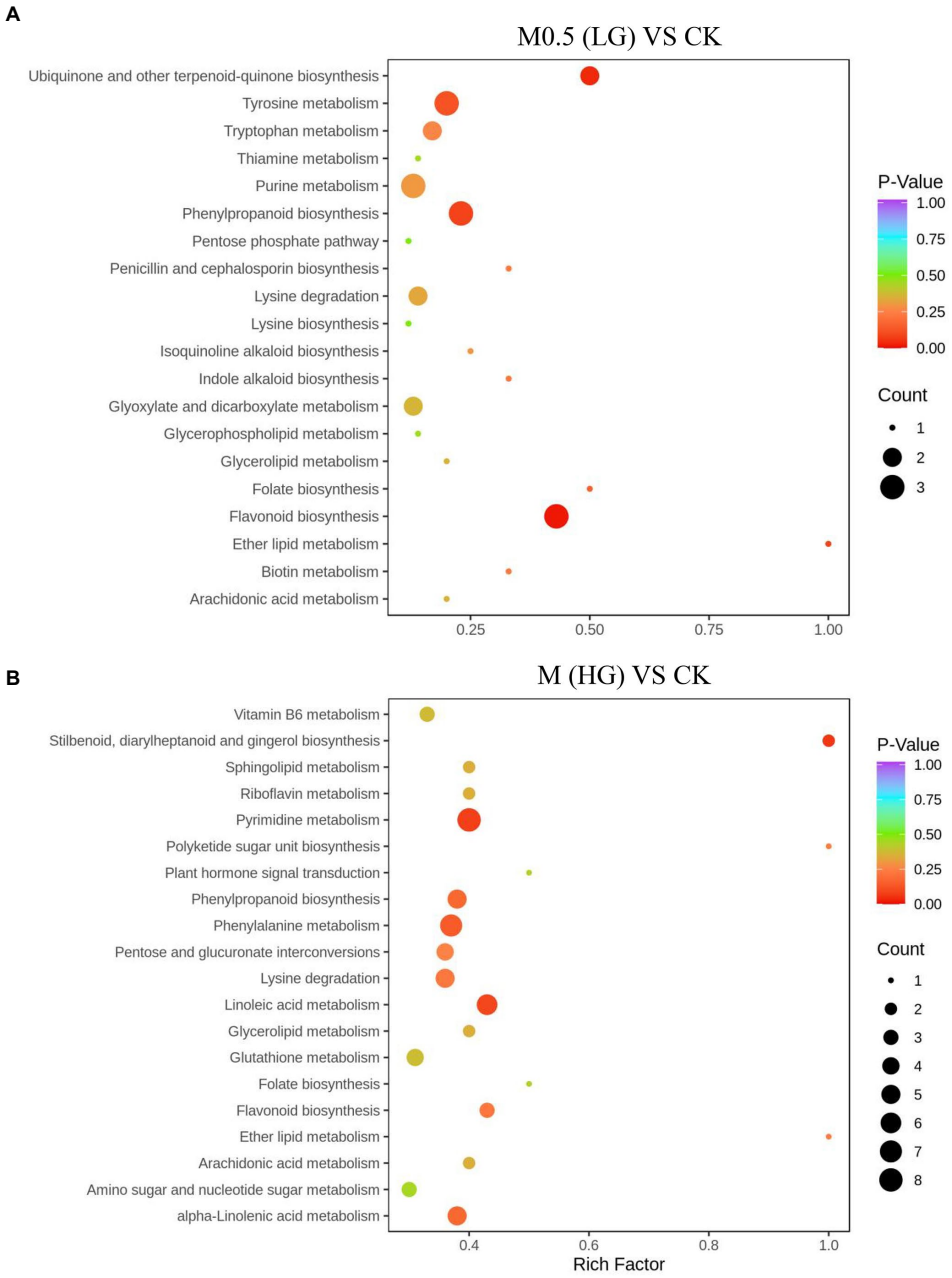
Top 20 enrichment pathways of DAMs from KEGG analysis. The two comparison groups were the DAMs of M0.5 (LG) compared with the control group **(A)** and M (HG) compared with the control group **(B)**. The X-axis is the rich factor, and the Y-axis represents the name of the pathway. The bubble size represents the number of DAMs involved. The bubble color indicates the enrichment degree of the pathway. All treatments are consistent with the above.

We annotated the DAMs using the KEGG database. The top 20 pathways with the highest enrichment in the two comparison groups are shown in Figure 4. Among them, flavonoid biosynthesis was enriched in the low-and high-treatment groups. Ubiquinone and other terpenoid-quinone biosynthesis pathways were enriched only in the low-treatment group (Figure 4A). Pyrimidine metabolism was associated with more enriched DAMs in the high treatment group (Figure 4B).

**Figure 5:**
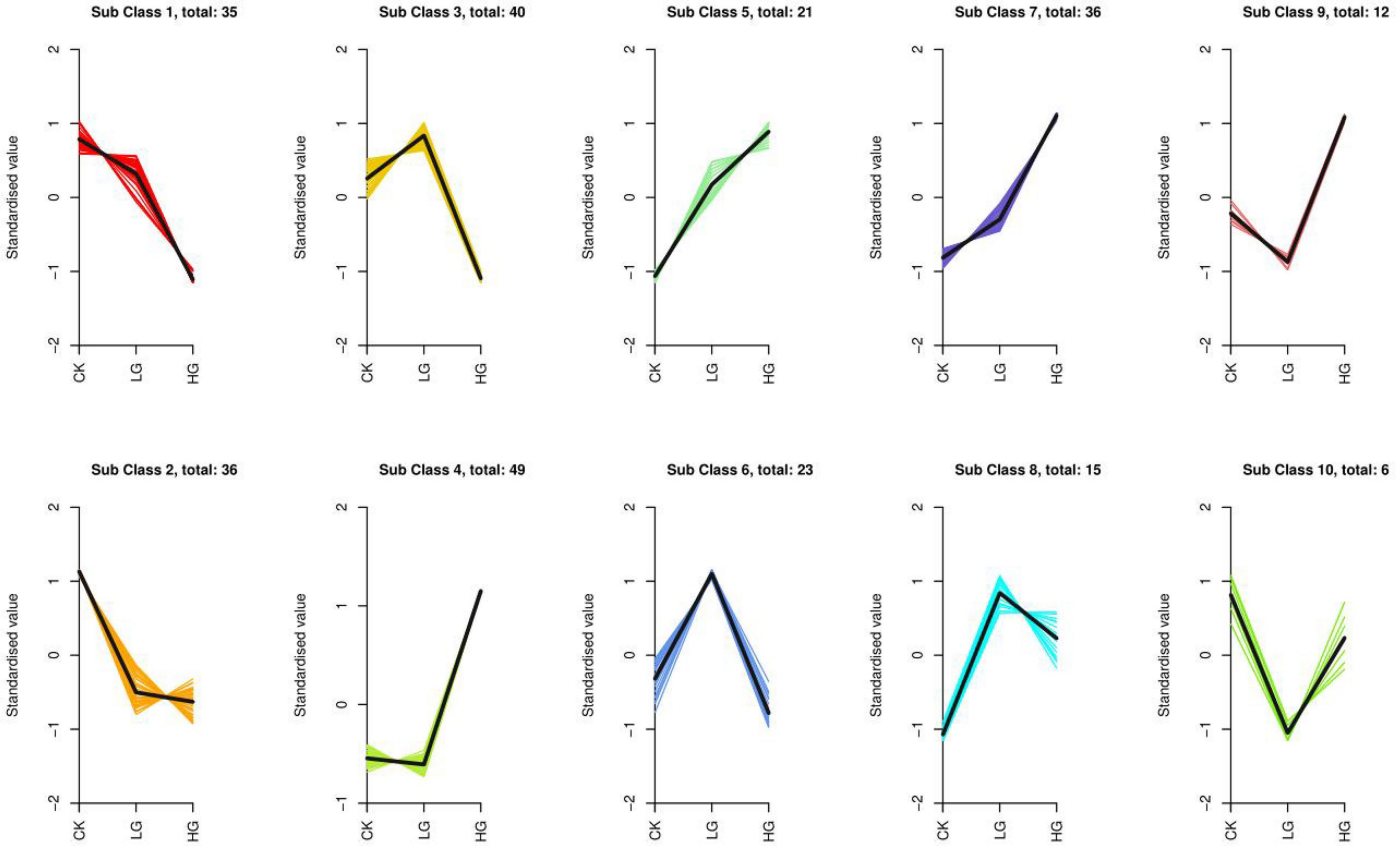
K-means cluster of variation trends of the relative content of metabolites in different groups. Three treatments were CK, LG (M0.5) and HG (M). All treatments are consistent with the above.

To evaluate the change trend of metabolites among the three groups, the relative contents of all different metabolites identified according to the screening criteria in all group comparisons were standardized using the Z Score, followed by K-means clustering analysis. The analysis results are shown in Figure 5. In different groups, the metabolites gradually increased from a low to high concentration, such as Sub Class 5 and Sub Class 7, with a total of 57 metabolites, most of which are alkaloids, phenolic acids, lipids, amino acids and derivatives, nucleotides and derivatives. The gradually decreasing metabolites included Sub Class 1 and Sub Class 2, with 71 metabolites. These substances mainly include organic acids, lipids, phenolic acids and flavonoids (Supplementary Table S2).

### Proteomics profile in response to different concentrations of *Alpinia officinarum* EO

**Figure 6:**
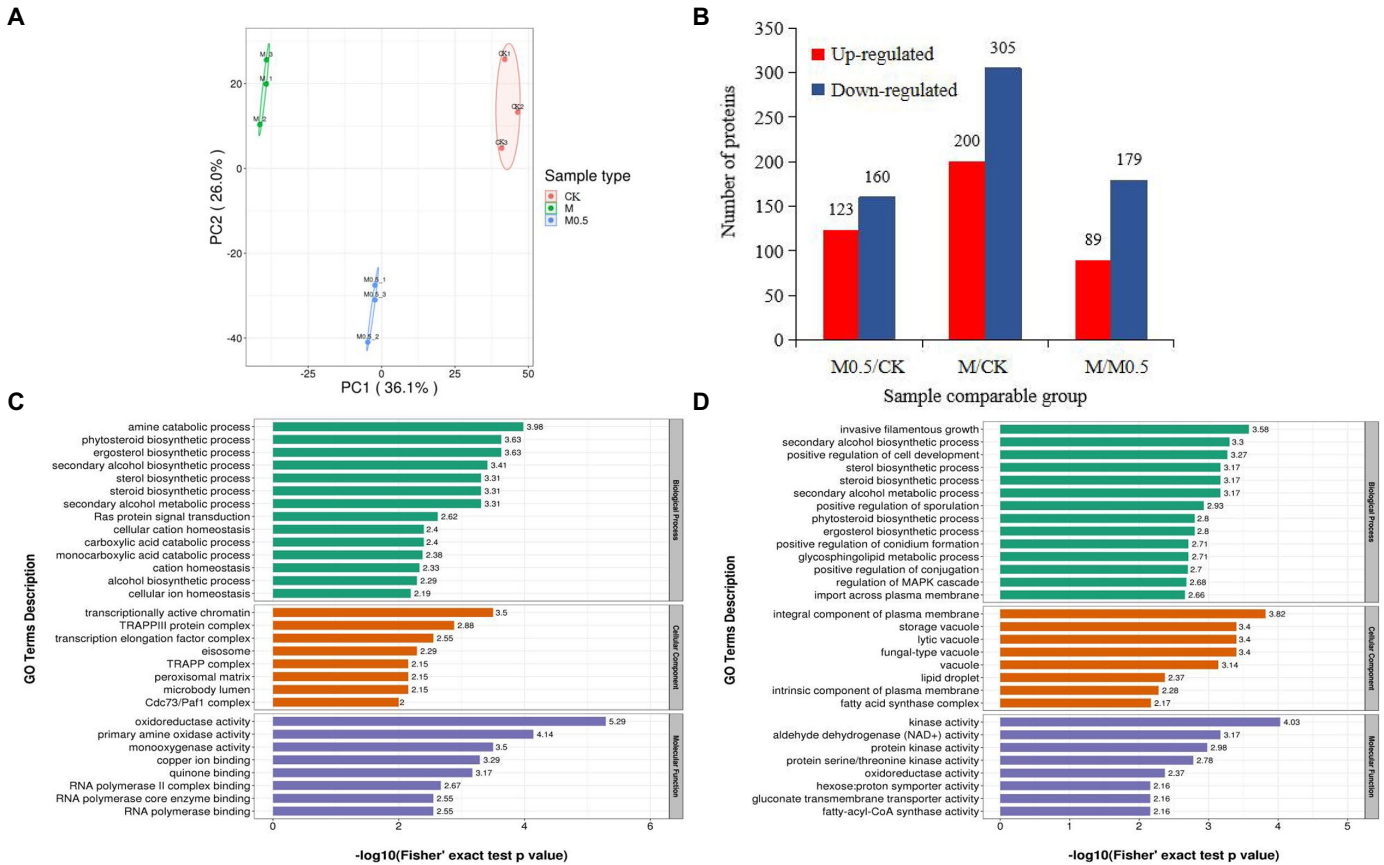
Number of enriched proteins and GO classification involved in specific biological processes in different comparisons. **(A)** Principal component analysis of protein quantification among 3 replicate samples. **(B)** Number of differentially expressed proteins (DEPs) identified in three comparision groups. **(C)** GO enrichment analysis of DEPs in M0.5 vs. CK **(C)** and in M vs. CK **(D)** including biological process, cellular component and molecular function. Green columns represented the categorization of biological process, orange columns represented the categorization of cellular component, and blue columns represented the categorization of molecular function.

We used 4D label-free proteomics to identify and quantify the proteins in control samples and samples treated with two different concentrations. The data analysis indicated that the total number of identified proteins was 4,688, 3,961 of which were quantifiable. The two-dimensional scatter diagram of protein quantitative principal component analysis between the three groups of repeated samples is shown in Figure 6A. The repeatability of the three groups of samples was good. A total of 283 differentially expressed proteins (DEPs) were identified in M0.5 vs. CK, 123 upregulated and 160 downregulated DEPs. Compared with CK, 200 upregulated DEPs and 305 downregulated DEPs were observed with the M treatment. Compared with M0.5, 89 upregulated and 179 downregulated DEPs were obtained with the M treatment (Figure 6B).

### Functional enrichment analysis of DEPs

The associations of proteins with specific biological processes were identified and divided into three major groups—cellular component (CC), molecular function (MF), and biological process (BP). In M0.5 vs. CK, the detected enriched proteins included CC (287), MF (129), and BP (396). The most enriched MF protein was catalytic activity (67), followed by binding (49). In CC, cell (105) and organelle (88) were highly enriched; in the BP category, cellular process showed the highest number of proteins (92), followed by metabolic process (78; Supplementary Figure S4A). In M vs. CK, MF, BP, and CC were enriched with 240, 778, and 568 proteins, respectively, and most of the proteins were enriched in BP. The catalog with the highest concentration of each component was the same as that in M0.5 vs. CK (Supplementary Figure S4B).

In M0.5 vs. CK, BP mainly included amine and carboxylic acid catabolic processes, suggesting that pathogens could maintain biological activity by regulating carbon and nitrogen metabolism under low concentration treatment. At the same time, CC and MF were mainly enriched in transcription-related protein regulating genes, indicating that under low concentration treatment, *F. oxysporum* could feedback the regulation of transcription factors to regulate the expression of stress-related genes to cope with stress. This finding is consistent with the low concentration of Fusarium not significantly changing the physiological indicators. Furthermore, the cell membrane, organelle membrane components and sterol synthesis were significantly increased, and biosynthesis inhibition regulation was decreased to resist the damage of EO on the cell membrane. The inhibition of positive regulation of conidium formation and DNA repair resulted in a significantly lower conidium germination rate (Figure 6C; Supplementary Table S3).

In M vs. CK, similar to the low concentration treatment, the damage of EOs to cell membranes was resisted by significantly increasing the plasma membrane composition, sterol synthesis and sphingolipid metabolism. High concentrations of EOs severely damaged the cell membranes, resulting in an enhanced transmembrane import process. At the same time, because of the enrichment in proteins related to invasive filamentous growth, positive regulation of cell development, sporulation, conidium formation and other biological processes decreased significantly, resulting in the spore germination, growth and development of *F. oxysporum* being seriously affected after high concentration treatment. The increase in various types of vacuolar and plasma membrane-associated proteins was also consistent with the degradation of organelles by autophagy observed by electron microscopy (Figure 2). A significant decrease in the activity of various kinases also indicates a significant decrease in biological activity (Figure 6D; Supplementary Table S4).

**Figure 7:**
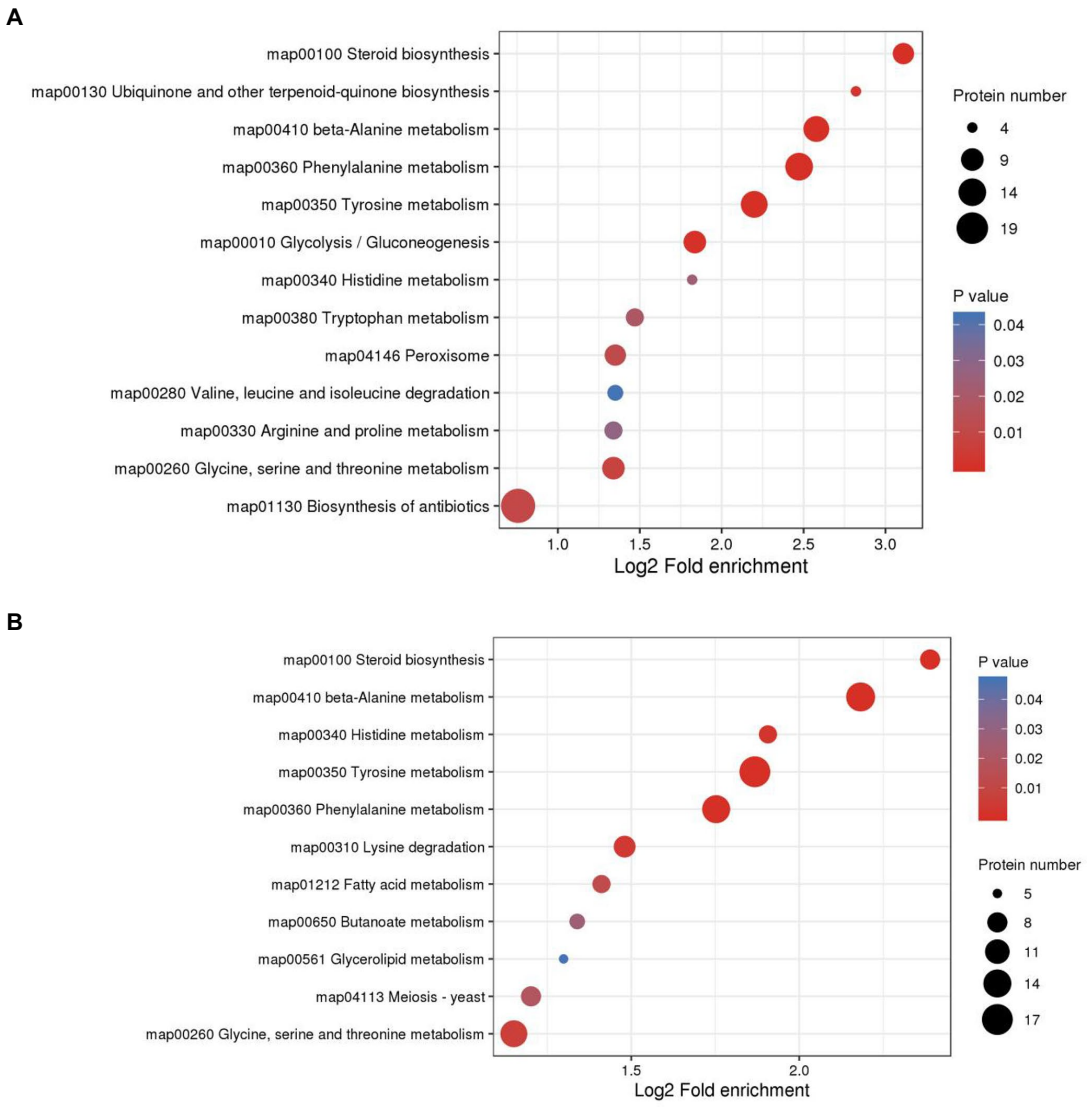
KEGG enrichment analysis was performed for each protein class and the enrichment results for different classifications were combined. The horizontal axis shows fold enrichment after Log2 transformation, the vertical axis shows the functional classification, the bubble size represents the number of proteins, and the bubble color represents the value of p of enrichment significance. **(A)** KEGG enrichment analysis of DEPs in M0.5 vs. CK. **(B)** KEGG enrichment analysis of DEPs in M vs. CK.

KEGG enrichment of differential proteins showed that in M0.5 vs. C, differential proteins were mainly enriched in amino acid-related pathways, carbon metabolism pathways, steroid-related synthesis pathways, antibiotic synthesis pathways and peroxisome intermediate pathways. This result also showed that after low-concentration treatment, *F. oxysporum* maintained biological activity primarily by regulating carbon and nitrogen metabolism, repaired membrane structures damaged by EOs by enhancing the steroid-related synthesis pathway, and enhanced the antibiotic synthesis pathway and peroxisome pathway to resist cell toxicity caused by EOs (Figure 7A; Supplementary Table S5). However, in M vs. C, although similar to the low concentration treatment, steroid and lipid metabolism were enhanced to resist damage to the membrane structure, and DEPs enriched in amino acid metabolism, sugar metabolism and the fatty acid metabolism pathway were significantly reduced at high concentrations, indicating that under high concentration treatment, the basic physiological functions of *F. oxysporum* were severely damaged. Additionally, meiosis-related proteins have also been sharply reduced, substantially hindering *F. oxysporum* reproduction. This finding is also consistent with the blocked spore germination and growth and development of *F. oxysporum* under high concentrations (Figure 7B; Supplementary Table S6). These results were consistent with the metabolomic results.

### Integrated proteomics and metabolome analysis

**Figure 8:**
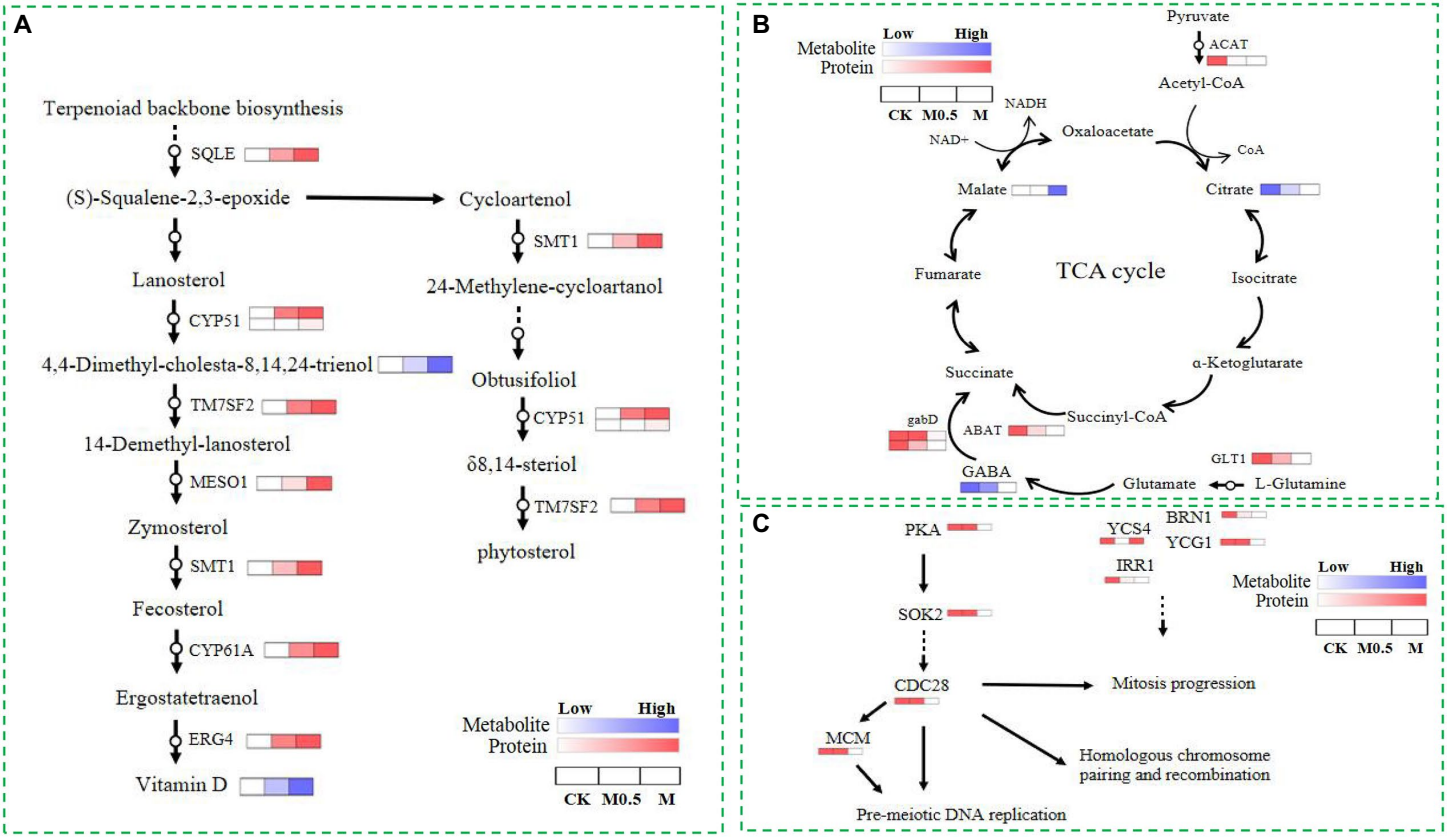
Differential proteins and metabolites involved in steroid biosynthesis **(A)**, the TCA cycle **(B)** and the cell cycle and meiosis **(C)** in CK, M0.5 and M.

As mentioned above, both low and high treatment concentrations were used to resist membrane structure destruction by increasing the function of steroid biosynthesis. The combined analysis of the proteome and metabolome showed that the expression of various catalytic enzymes in this pathway increased significantly after treatment, such as squalene monooxygenase (SQLE), sterol 14alpha-demethylase (CYP5, CYP61A), delta 14-sterol reductase (TM7SF2, ERG4), methylsterol monooxygenase (MESO1), and sterol 24-C-methyltransferase (STM1), and the expression of these enzymes increased with the concentration of treatment, as did some intermediate metabolites in this pathway (Figure 8A). The above results indicated that the expression of genes related to these enzymes was possibly stimulated by the EO, and the function was reflected by feedback into steroid biosynthesis.

Additionally, EOs inhibit the expression of cohesin complex subunit SA-1/2 (IRR1) and cohesion complex subunit (YCS4, BRN1, YCG1). However, a high concentration of EO further inhibits cyclin-dependent kinase (CDC28) and DNA replication licensing factor (MCM) to disrupt the cell cycle and meiosis, affecting cell division. Finally, the spore production and germination of *F. oxysporum* spores treated with EO were inhibited. In our study, the TCA cycle was regulated primarily by influencing collateral, affecting the normal growth of *F. oxysporum* (Figures 8B,C). After EO treatment, the expression of glutamate synthase (GLT1) in the glutamate metabolic pathway is inhibited, and gamma-aminobutyric acid (GABA) is reduced. Additionally, decreased expression of downstream 4-aminobutyrate aminotransferase (ABAT) and succinate-semialdehyde dehydrogenase (gabD) further affects the metabolism of succinate in the TCA cycle. However, the expression of acetyl-CoA C-acetyltransferase (ACAT) in the pyruvate metabolism pathway decreased with increasing L-malic acid content. The expression of these proteins and changes in the primary metabolites indicated that the TCA cycle changed after volatile oil treatment.

### Analysis of relative content of metabolites and verification of DEPs *via* qPCR

Relative content of vitamin D, 4.4-Dimethyl-cholesta-8.14.24-trienol and matate were increased under the EO treatment when compared with control, whereas GABA and citrate were decreased under the EO treatment in TCA cycle (Supplementary Figure S5A). The proteins involved in steroid biosynthesis (SQLE，CYP51，TM7SF2，MESO1，SMT1，CYP61A), the TCA cycle (GLT1，ABAT，GABD，ACAT) and the cell cycle and meiosis (PKA，SOK2，CDC28，MCM，YCS4，BRN1，YCG1，IRR1) were further confirmed by qRT-PCR. As shown in Supplementary Figure S5, the expression levels of genes related to steroid biosynthesis were upregulated after treatment of EO (Supplementary Figure S5B). Genes associated with TCA cycle and the cell cycle and meiosis of *F. oxysporum* were downregulated after treatment of EO (Supplementary Figures S5C,D). Overall, the trend of gene expression was consistent with the results of the proteomic data.

## Discussion

The cell membrane is primarily composed of lipids (mainly phospholipids), proteins and sugars and is considered the first barrier that separates a cell from the external environment to ensure the relative stability of the internal environment (Zhang and Rock, 2008). The change in membrane permeability was evaluated by determining the extracellular conductivity of *F. oxysporum* treated with and without EO (Figure 1B). Under the effect of the MIC concentration of EO, the cell membrane conductivity increased gradually with the extension of treatment time after 2.5 h, but no significant difference was found between the low concentration and control. The fungal membrane permeability was changed, and the spore no longer grew. Inhibition of the positive regulation of conidium formation and DNA repair (Figure 6C; Supplementary Table S2) resulted in a significantly lower spore germination rate (Figure 1A). Subsequently, mycelium growth was also affected, resulting in a significant decrease in both the fresh and dry weight (Figures 1C,D). Because the cell membrane was destroyed, the cell contents, including reducing sugars and soluble proteins, leaked out of the cell from the inside (Figures 1E,F). These results indicated that the EO could inhibit fungal growth and change the conductivity in the medium. The integrity of the mycelia might have been destroyed, and the structure of the phospholipid bilayer was disturbed by *A. officinarum* EO.

Ergosterol is an essential component of the microbial cell membrane and plays a critical role in ensuring cell membrane integrity, membrane binding enzyme activity, membrane fluidity, cell viability and cell material transport. According to a previous report, ergosterol maintains the structural integrity of the yeast membrane under stress environmental conditions (Dickey et al., 2009; Villares et al., 2014). In our study, functional enrichment analysis of DEPs showed that the cell membrane, organelle membrane components and sterol synthesis were significantly increased, and biosynthesis inhibition regulation was decreased to resist the damage of EO on the cell membrane in M0.5 vs. CK (Figure 6C; Supplementary Table S3). Furthermore, the antibiotic synthesis and peroxisome pathways were enhanced to resist cell toxicity caused by EOs (Figure 7A; Supplementary Table S5). Under high EO concentrations, in addition to matching the response at low concentrations, the transmembrane import process was enhanced (Figure 7B; Supplementary Table S6). The main volatile components in *A. officinarum* EO were eucalyptol (45.437%), (+)-α-terpineol (9.79%), camphene (5.702%), (+/−)-α-Pinene (2.243%) and d-camphor (1.998%; Supplementary Figure S2). Camphor and eucalyptol are bioactive terpenoids, and their antifungal properties have been explored previously. Eucalyptol exhibited anti-biofilm activity against mature and developing biofilms of *Candida albicans* and *Candida glabrata* along with their clinical isolates and altered mitochondrial membrane potential (Gupta et al., 2021). Camphor showed excellent antifungal activity by blocking the hyphal transition along with its impact on genes encoding efflux pumps (*CDR1* and *CDR2*) and ergosterol biosynthesis (*ERG11*; Ivanov et al., 2021). The antibacterial activities of α-terpineol occurred in a concentration-dependent manner by destroying the cell membrane and wall, resulting in cell death. Release of nucleic acids, proteins and alkaline phosphatase (AKP), along with a decrease in membrane potential, was observed (Huang et al., 2020). In this experiment, mycelial and cell morphology were seriously damaged after M0.5 and M treatment for 24 h (Figure 2), indicating that these monomeric compounds could also affect the structure of the *F. oxysporum* cell wall and cell membrane. Under low concentration treatment, *F. oxysporum* regulates the expression of stress-related genes by feedback regulating transcription factors to cope with stress. This finding is consistent with the low concentration of *F. oxysporum* not significantly changing the physiological indicators. At the same time, it can resist the destruction of the cell membrane by significantly increasing the components of the cell membrane and organelle membrane, increasing sterol synthesis and reducing the inhibitory regulation of biosynthesis.

The ubiquitous tricarboxylic acid cycle (TCA) is the core process of all biological matter and energy metabolism, regulating crucial physiological activities of cells through the production of “energy currency” ATP and metabolic intermediates. The TCA cycle is a central pathway for the metabolism of carbon sources, lipids and amino acids and provides a major energy source for the cell under aerobic conditions. The TCA cycle regulates the hyphal development of *Candida albicans*, influencing its pathogenicity (Tao et al., 2017). KEGG pathway analysis of proteomics and metabolomics revealed that most of the proteins and metabolites were enriched in amino acid metabolism, phenylalanine metabolism, lipid metabolism and nucleotide metabolism and were mostly associated with the TCA cycle in amino acid metabolism, indicating that a wide metabolic disorder was caused after EO treatment, resulting in TCA cycle disorders (Figures 4, 7). At low concentrations, *F. oxysporum* maintained its biological activity mainly by regulating carbon and nitrogen metabolism, repairing the membrane structure damaged by EOs by enhancing steroid-related synthesis pathways, and resisting cell toxicity caused by EOs by enhancing antibiotic synthesis pathways and peroxisome pathways. At high concentrations, although similar to low concentration processing, such as the enhancement of steroids and lipid metabolism to resist damage to membrane structure, most of the enrichment in amino acid metabolism and sugar metabolism and the fatty acid metabolism pathway of DEPs was significantly reduced, suggesting that the basic physiological functions of *F. oxysporum* were severely damaged under high concentration treatment. This phenomenon can be observed using the physiological indicators in Figure 1 and electron microscope in Figure 2.

The PKA pathway is critical in initiating the signaling processes that promote cell growth and division (Busti et al., 2010). The MCM complex is defined as being licensed for replication in eukaryotes. When the MCM complex (DNA replication licensing factor) is loaded on chromatin, the replication originates (Nishitani and Lygerou, 2004). MCM is kept inactive during G1, and it initiates replication after being activated in S phase (Wei and Zhao, 2016). A previous study showed that the chromosome condensation factor Brn1p is required for chromatid separation in mitosis. Mutant brn1 cells show a defect in mitotic chromosome condensation and sister chromatid separation and segregation in anaphase (Huang et al., 2020). BRN1 in *Saccharomyces cerevisiae* performs an essential function during a period of the cell cycle when chromosome condensation is established and maintained. It can influence multiple aspects of chromosome transmission and dynamics (Lavoie et al., 2000). YCS4 function is required to localize DNA topoisomerase I and II to chromosomes and is associated with chromatin throughout the cell cycle (Gupta et al., 2021). Ycs4 is required for efficient double-strand break (DSB) formation and establishing homolog bias at the early stage of meiotic prophase I, which allows efficient formation of interhomolog recombination products (Hong et al., 2015). The levels of Ycg1, the Cap-G subunit of budding yeast condensin, limit condensin function during the cell cycle (Doughty et al., 2016). In our study, PKA pathway-related proteins and meiosis-related proteins were sharply decreased, which disrupted the cell cycle and meiosis, thus affecting cell division. The significant increase in nucleotides and their derivatives indicated that genomic instability in treated *F. oxysporum* cells resulted in the decomposition of DNA or RNA, while cell viability was greatly reduced after high-concentration treatment, and secondary metabolites such as flavonoids were sharply reduced. The propagation of *F. oxysporum* was substantially hindered, resulting in the inhibition of spore germination and development of *F. oxysporum* at high concentrations (Figures 1, 8).

## Conclusion

**Figure 9:**
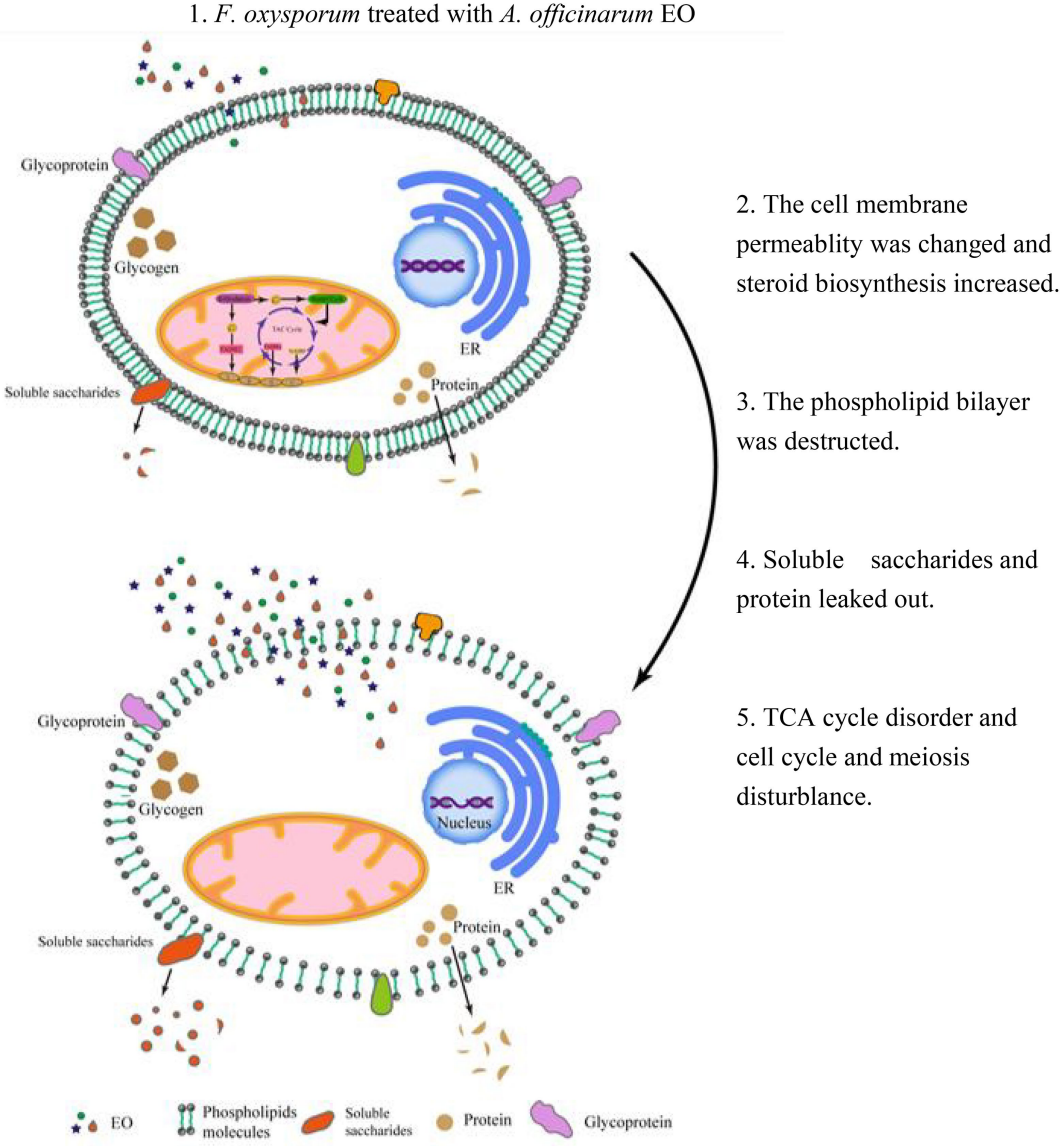
The mechanism overview of *A. officinarum* EO on *F. oxysporum*.

*A. Officinarum* EO contains various lipid soluble substances that can easily penetrate the phospholipid bilayer membrane of *F. oxysporum*, destroy the membrane of fungi, enhance membrane permeability, and cause the extravasation of cell contents. At low concentrations, *F. oxysporum* can resist the damage of *A. Officinarum* EO to the membrane system by enhancing the synthesis of sterols. At the same time, it can maintain biological activity by regulating carbon and nitrogen metabolism and resist cell toxicity caused by *A. Officinarum* EO by enhancing the antibiotic synthesis pathway and peroxisome pathway. Under the high concentration of *A. Officinarum* EO, although *F. oxysporum* still tried to resist damage to the membrane structure by enhancing steroid and lipid metabolism, the activity of various kinases was significantly reduced, and the basic physiological functions were severely damaged. Additionally, meiosis-related proteins were sharply reduced, considerably hindering the reproduction of *F. oxysporum* (Figure 9). Therefore, the damage of *A. Officinarum* EO to *F. oxysporum* is concentration dependent and time dependent, and the damage of *A. Officinarum* EO to *F. oxysporum* is multitargeted because of the coaction of various chemical components of *A. Officinarum* EO.

## Data availability statement

The original contributions presented in the study are included in the article/Supplementary material, further inquiries can be directed to the corresponding author.

## Author contributions

XD designed the experiment and wrote the paper. XD, X-YL, and Y-YH analyzed the data. X-YL, Y-YH, JY, and T-TL performed the experiments. F-RX, H-PW, J-NL, C-HW, and Y-HZ commented the paper. All authors contributed to the article and approved the submitted version.

## Funding

This work was funded by the Yunnan Provincial Science and Technology Department-Applied Basic Research Joint Special Funds of Yunnan University of Chinese Medicine [2019FF002(-003)], National Natural Science Foundation of China (82060683) and the Open Project of Hubei Key Laboratory of Wudang Local Chinese Medicine Research (Hubei University of Medicine; WDCM2022014).

## Conflict of interest

The authors declare that the research was conducted in the absence of any commercial or financial relationships that could be construed as a potential conflict of interest.

## Publisher’s note

All claims expressed in this article are solely those of the authors and do not necessarily represent those of their affiliated organizations, or those of the publisher, the editors and the reviewers. Any product that may be evaluated in this article, or claim that may be made by its manufacturer, is not guaranteed or endorsed by the publisher.

## Supplementary material

The Supplementary material for this article can be found online at: https://www.frontiersin.org/articles/10.3389/fmicb.2022.1031474/full#supplementary-material

## Footnotes

^1^ www.shimadzu.com.cn/

^2^ www.appliedbiosystems.com.cn/

^3^ www.r-project.org/

^4^ http://www.ebi.ac.uk/GOA
