# Supplementary material for: Integrated physiological, metabolomic, and proteome analysis of Alpinia officinarum Hance essential oil inhibits the growth of Fusarium oxysporum of Panax notoginseng
Source: Front Microbiol. 2022 Nov 16;13:1031474. doi: 10.3389/fmicb.2022.1031474 (PMC9724623; doi:10.3389/fmicb.2022.1031474)
Supplement: Supplementary file 1 [file Table_1.docx]

Table S1 Main chemical constituents and contents of the essential oil of *Alpinia officinarum* Hance

| NO. | RT | Component | CAS | RI | Content（%） |
| --- | --- | --- | --- | --- | --- |
| 1 | 6.032 | [Isobutyl isobutyrate](https://pubchem.ncbi.nlm.nih.gov/compound/7351) | 97-85-8 | 1118.087698 | 0.563±0.024 |
| 2 | 6.432 | α-Pinene | 80-56-8 | 1135.946364 | 2.243±0.080 |
| 3 | 6.642 | Comphene | 79-92-5 | 1146.776689 | 5.702±0.256 |
| 4 | 7.145 | 2-Methylbutyl isobutyrate | 2445-69-4 | 1100.727179 | 0.279±0.033 |
| 5 | 7.41 | α-Terpinen； | 99-86-5 | 1178.287777 | 0.450±0.023 |
| 6 | 7.728 | Eucalyptol | 470-82-6 | 1186.384734 | 45.437±1.596 |
| 7 | 8.09 | γ-Terpinen | 99-85-4 | 1201.749838 | 0.406±0.016 |
| 8 | 8.728 | 4-Carene | 29050-33-7 | 1213.480233 | 1.381±0.068 |
| 9 | 9.43 | Fenchol | 1632-73-1 | 1234.154245 | 0.617±0.015 |
| 10 | 10.138 | β-Terpineol | 138-87-4 | 1256.902139 | 0.159±0.004 |
| 11 | 10.381 | D-Camphor | 464-49-3 | 1279.844459 | 1.998±0.076 |
| 12 | 10.51 | Camphene Hydrate | 465-31-6 | 1287.71873 | 0.333±0.026 |
| 13 | 10.705 | Isoborneol | 124-76-5 | 1291.898898 | 0.983±0.033 |
| 14 | 10.948 | Borneol | 507-70-0 | 1304.080747 | 1.401±0.047 |
| 15 | 11.234 | 4-Carvomenthenol | 562-74-3 | 1310.288691 | 1.680±0.050 |
| 16 | 11.639 | (+)-α-Terpineol | 7785-53-7 | 1319.079661 | 9.79±0.348 |
| 17 | 11.828 | γ-Terpineol | 586-81-2 | 1323.182114 | 0.284±0.011 |
| 18 | 12.617 | Fenchylacetate | 13851-11-1 | 1340.308227 | 0.277±0.007 |
| 19 | 13.552 | Benzylacetone | 2550-26-7 | 1360.60343 | 0.231±0.006 |
| 20 | 15.48 | Bornyl Acetate | 76-49-3 | 1401.828479 | 0.111±0.003 |
| 21 | 17.614 | Butyl Benzoate | 136-60-7 | 1436.359223 | 0.140±0.001 |
| 22 | 21.59 | Phenethyl Isobutyrate | 103-48-0 | 1500.570367 | 0.406±0.015 |
| 23 | 22.87 | Isocaryophyllene | 118-65-0 | 1517.548747 | 0.359±0.013 |
| 24 | 24.529 | Trans-α-Bergamotene | 13474-59-4 | 1539.554318 | 0.197±0.008 |
| 25 | 26.225 | α-Humulene | 6753-98-6 | 1562.05067 | 0.204±0.036 |
| 26 | 27.743 | γ-Muurolene | 30021-74-0 | 1582.185966 | 0.236±0.016 |
| 27 | 28.137 | 2-Carboxymethyl-3-Hexylmaleic Acid Anhydride | 39212-21-0 | 1587.412124 | 0.228±0.042 |
| 28 | 28.288 | 2-Phenylethyl 2-Methylbutanoate | 24817-51-4 | 1589.415042 | 0.297±0.019 |
| 29 | 28.694 | (-)-β-Selinene | 17066-67-0 | 1594.800371 | 0.320±0.030 |
| 30 | 29.131 | Valencene | 4630-07-3 | 1600.529786 | 0.107±0.004 |
| 31 | 29.812 | α-Farnesene | 502-61-4 | 1608.54721 | 0.139±0.004 |
| 32 | 30.06 | β-Bisabolene | 495-61-4 | 1611.466918 | 0.122±0.010 |
| 33 | 30.779 | (-)-γ-Cadinene | 39029-41-9 | 1611.466918 | 0.553±0.021 |
| 34 | 31.405 | δ-Amorphene | 16729-01-4 | 1627.301625 | 0.230±0.012 |
| 35 | 32.561 | (4aR,8aS)-4a-Methyl-1-methylene-7-(propan-2-ylidene)decahydronaphthalene | 58893-88-2 | 1640.911231 | 0.204±0.018 |
| 36 | 33.123 | α-Calacorene | 21391-99-1 | 1647.527667 | 0.259±0.013 |
| 37 | 39.314 | Epicubenol | 19912-67-5 | 1719.419868 | 0.191±0.004 |
| 38 | 40.794 | γ-Eudesmol | 1209-71-8 | 1635.995072 | 0.158±0.008 |
| 39 | 41.615 | Bicyclo[4.4.0]dec-1-ene, 2-isopropyl-5-methyl-9-methylene- | 150320-52-8 | 1745.189831 | 0.145±0.033 |
| 40 | 42.863 | α-Cadinol | 481-34-5 | 1759.16676 | 0.288±0.008 |
