# Supplementary figures and images for: Integrated physiological, metabolomic, and proteome analysis of Alpinia officinarum Hance essential oil inhibits the growth of Fusarium oxysporum of Panax notoginseng
Source: Front Microbiol. 2022 Nov 16;13:1031474. doi: 10.3389/fmicb.2022.1031474 (PMC9724623; doi:10.3389/fmicb.2022.1031474)

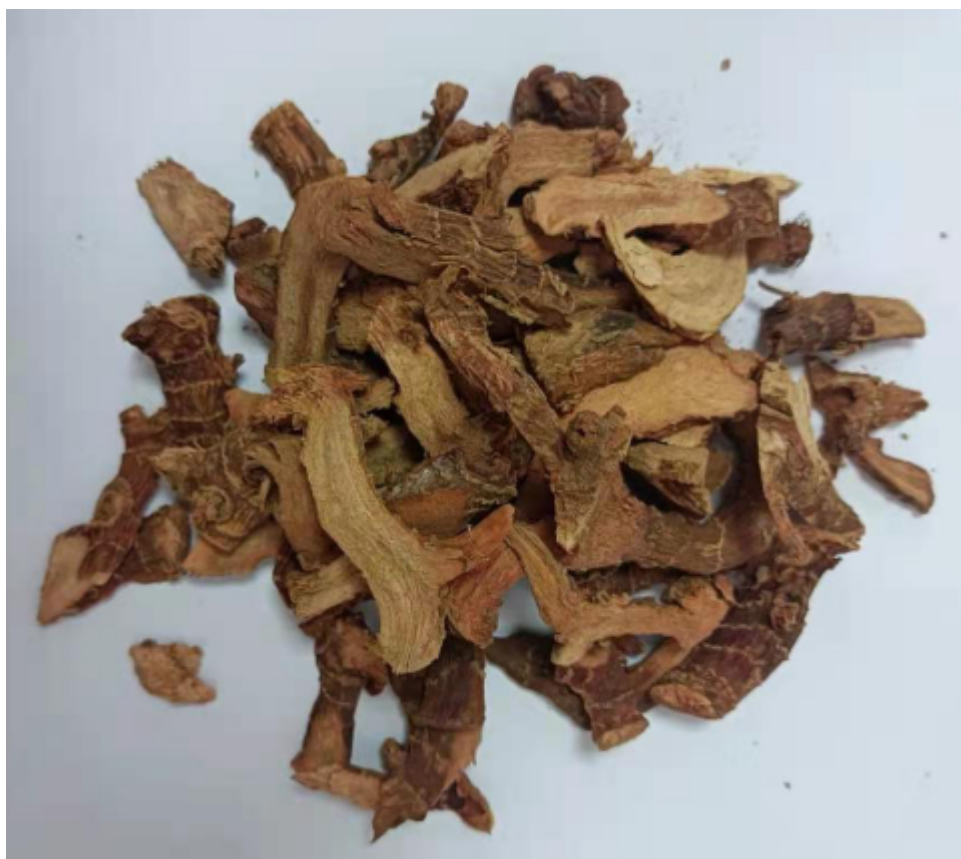

855

856

857 Fig. S1 Raw materials of *Alpinia officinarum* Hance

Supplement: Supplementary file 8 [file Image_1.pdf]
