# Supplementary material for: Integrated physiological, metabolomic, and proteome analysis of Alpinia officinarum Hance essential oil inhibits the growth of Fusarium oxysporum of Panax notoginseng
Source: Front Microbiol. 2022 Nov 16;13:1031474. doi: 10.3389/fmicb.2022.1031474 (PMC9724623; doi:10.3389/fmicb.2022.1031474)
Supplement: Supplementary file 9 [file Image_2.pdf]

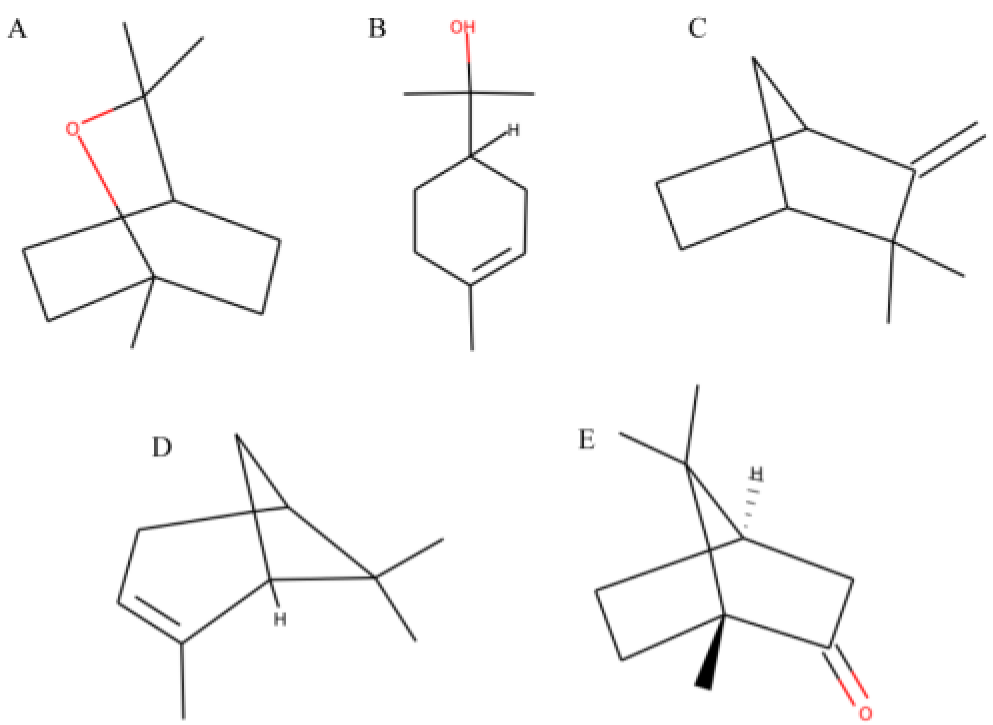

Fig. S2 Structures of the five main components of EO from *Alpinia officinarum* Hance.

(A) Eucalyptol; (B) (+)-alpha-terpineol; (C) camphene; (D) (+/-)-alpha-pinene; (E) D-camphor.
