# Supplementary material for: Integrated physiological, metabolomic, and proteome analysis of Alpinia officinarum Hance essential oil inhibits the growth of Fusarium oxysporum of Panax notoginseng
Source: Front Microbiol. 2022 Nov 16;13:1031474. doi: 10.3389/fmicb.2022.1031474 (PMC9724623; doi:10.3389/fmicb.2022.1031474)
Supplement: Supplementary file 10 [file Image_3.pdf]

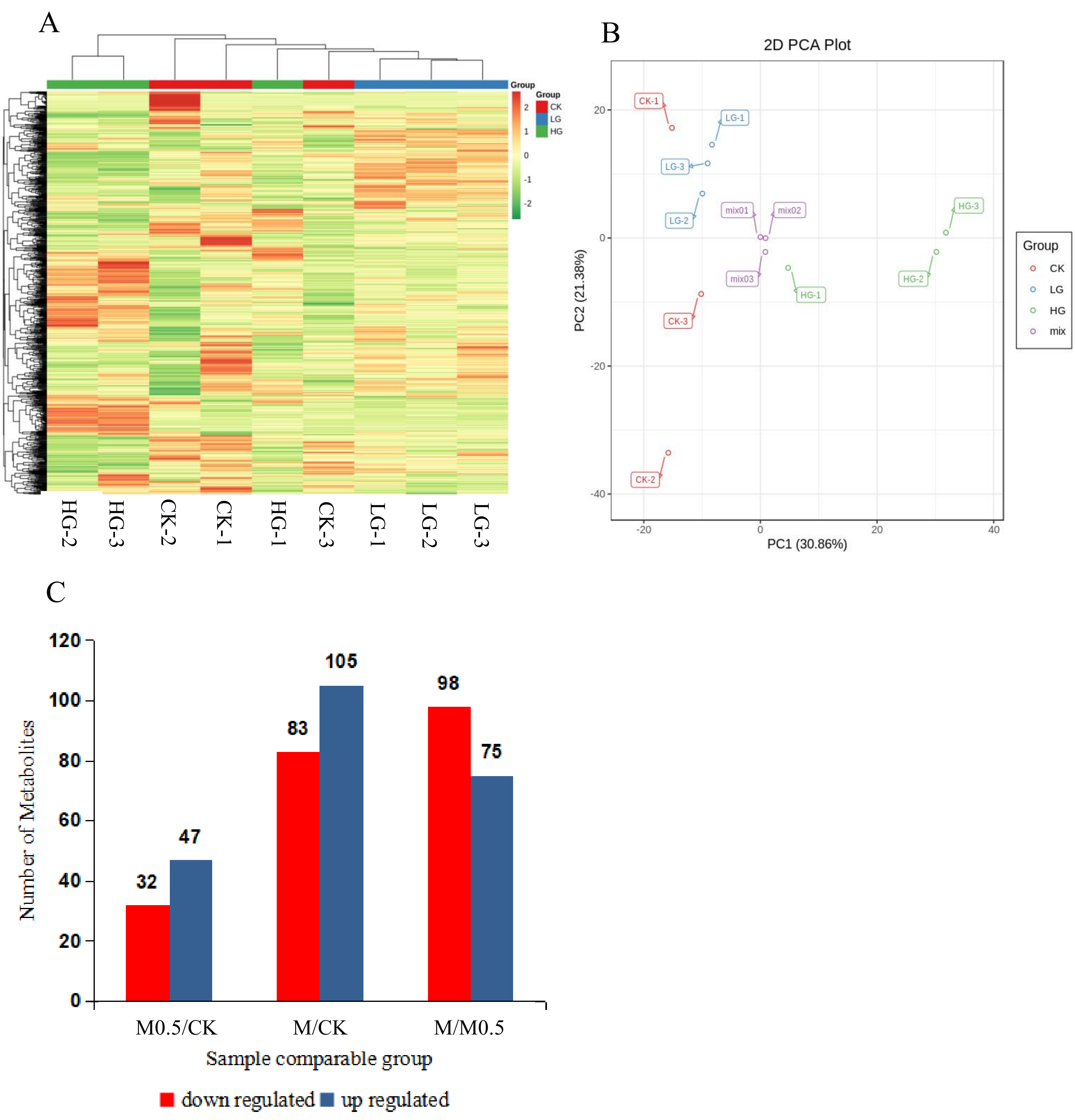

Fig. S3. Quality control of the metabolomics data.

(a) Heatmap of the results of clustering analysis of DAMs.

(b) PCA of DAMs.

(c) Number of DAMs in the three groups
