# Supplementary material for: Integrated physiological, metabolomic, and proteome analysis of Alpinia officinarum Hance essential oil inhibits the growth of Fusarium oxysporum of Panax notoginseng
Source: Front Microbiol. 2022 Nov 16;13:1031474. doi: 10.3389/fmicb.2022.1031474 (PMC9724623; doi:10.3389/fmicb.2022.1031474)
Supplement: Supplementary file 11 [file Image_4.pdf]

A

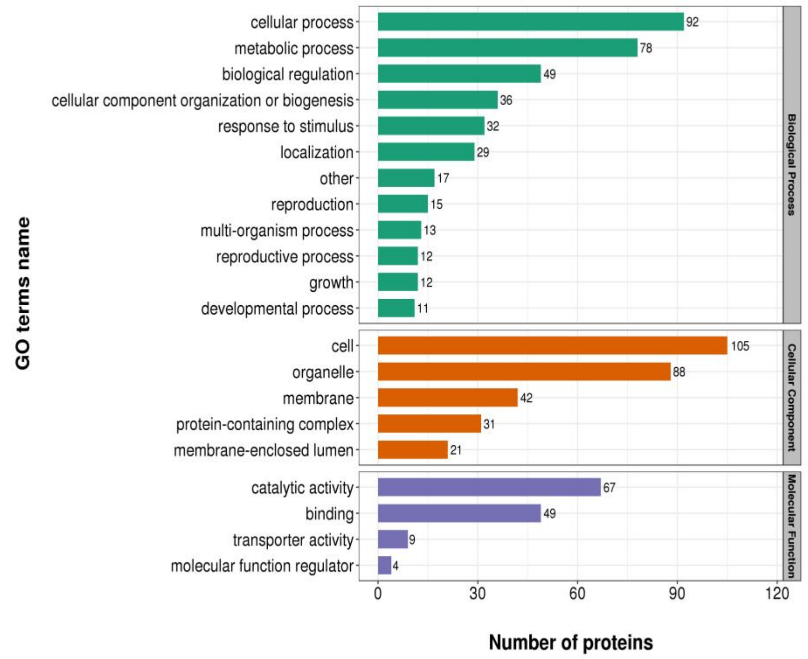

B

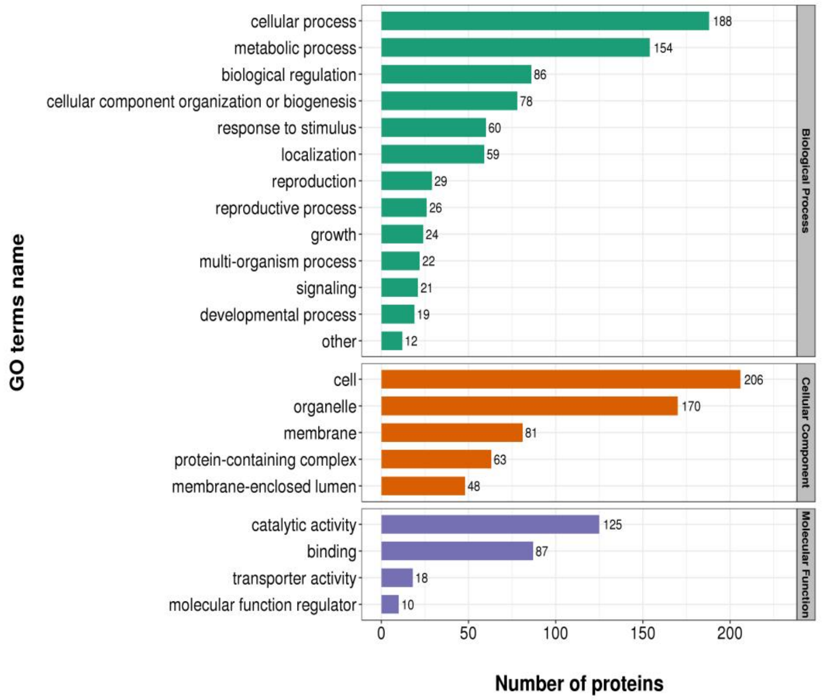

Fig. S4. Number of enriched proteins and GO classification involved in specific biological processes in different comparisons in the two groups. (a) Number of identified proteins and significantly enriched proteins with $P < 0.05$ involved in three categories, including biological process, cellular component and molecular function, in M0.5 vs. CK. (b) Number of different GO

terms in three categories in M vs. CK.
