# Supplementary material for: Integrated physiological, metabolomic, and proteome analysis of Alpinia officinarum Hance essential oil inhibits the growth of Fusarium oxysporum of Panax notoginseng
Source: Front Microbiol. 2022 Nov 16;13:1031474. doi: 10.3389/fmicb.2022.1031474 (PMC9724623; doi:10.3389/fmicb.2022.1031474)
Supplement: Supplementary file 12 [file Image_5.pdf]

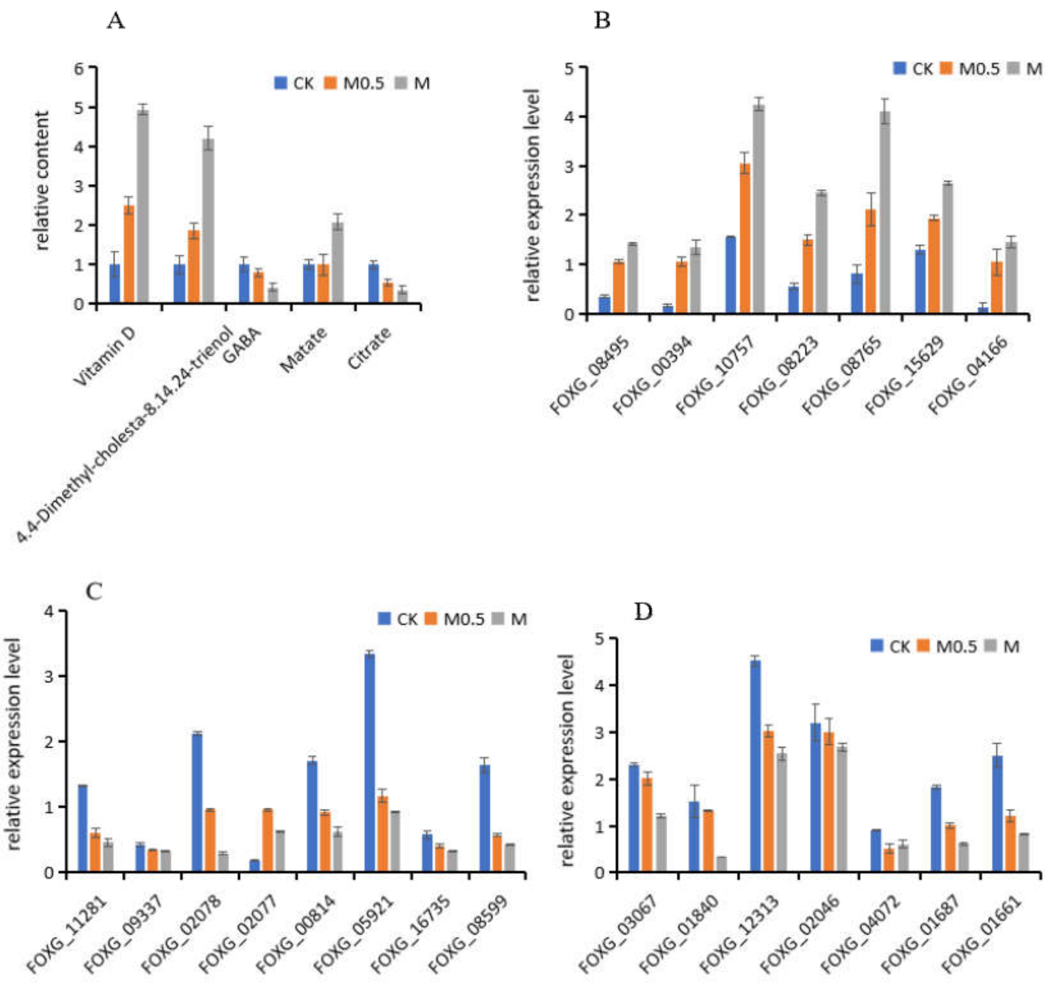

Fig. S5. The relative content of metabolites (A) and the expression of genes in the three pathways (B-D). Vitamin D and 4.4-Dimethyl-cholesta-8.14.24-trienol involved in steroid biosynthesis; GABA, matate and citrate involved in the TCA cycle. The expression of genes (*FOXG_0849*, *FOXG_00394*, *FOXG_10757*, *FOXG_08223*, *FOXG_08765*, *FOXG_15629* and *FOXG_04166*) involved in steroid biosynthesis (B), genes expression (*FOXG_11281*, *FOXG_09337*, *FOXG_02078*, *FOXG_02077*, *FOXG_00814*, *FOXG_05921*, *FOXG_16735*, *FOXG_08599*) invovled in TCA cycle (C) and genes expression (*FOXG_03067*, *FOXG_01840*, *FOXG_12313*, *FOXG_02046*, *FOXG_04072*, *FOXG_01687*, *FOXG_01661*) involved in the cell cycle and meiosis (D) were determined through qRT-PCR. Three treatments were consistent with above.
